# Elektrokonvulsionstherapie bei jahrzehntelang therapieresistenter schizoaffektiver Störung: Remission und Wiedererlangung der Einwilligungsfähigkeit

**DOI:** 10.1007/s00115-026-02013-x

**Published:** 2026-09-07

**Authors:** Guillermo Calvi, Dirk Haufe, Pavlo Lopatnov, Peter Prager, Johannes Petzold, Maik Spreer

**Affiliations:** 1 Klinik und Poliklinik für Psychiatrie und Psychotherapie, Universitätsklinikum Dresden, Technische Universität Dresden, Fetscherstraße 74, 01307 Dresden, Deutschland; 2 Klinik für Anästhesiologie und Intensivtherapie, Universitätsklinikum Dresden, Technische Universität Dresden, Dresden, Deutschland

## Fallbericht

### Anamnese und Verlauf

Bei einer 54-jährigen Patientin besteht seit fast 4 Jahrzehnten eine schizoaffektive Störung mit überwiegend schizomanem Verlauf. Aufgrund psychotischer Dekompensationen kam es in den 15 Jahren vor der hier berichteten Behandlung zu mehr als 10 stationären Aufenthalten von teils mehrmonatiger Dauer sowie einer zeitweisen Unterbringung in einer geschützten soziotherapeutischen Wohnform. Trotz multipler pharmakologischer Therapieversuche, darunter über 15 Monate Clozapin in therapeutisch wirksamen Serumspiegeln sowie Hochdosis- und Kombinationsstrategien typischer und atypischer Antipsychotika, wurde keine anhaltende Remission erreicht (Tab. 1). Vor Einleitung einer Elektrokonvulsionstherapie (EKT) befand sich die Patientin über 9 Monate auf einer geschützten Akutstation.

**Tab. 1:** Krankheits- und Behandlungsverlauf

Zeitraum	Phase	Behandlungsstrategie	Klinisches Ergebnis
1988	Erstmanifestation psychotische Episode	Typische Antipsychotika, z. B. Haloperidol	Partielle Remission
1990–2005	Rezidivierende Episoden	Sequenziell verschiedene typische und atypische Antipsychotika	Rezidivierende Dekompensationen
2006–2015	Zunehmende Chronifizierung	Mono- und Kombinationstherapien, Polypharmazie, Hochdosisstrategien, u. a. Olanzapin, Quetiapin, Clozapin (02/2013–06/2013 und 08/2013–05/2014), Flupentixol, Zuclopenthixol, Pimozid	Nur Teilstabilisierung; zeitweise geschützte Wohnform
2016–2024	Ausgeprägte Therapieresistenz		Persistierende affektive und psychotische Symptomatik
2025	Therapieresistente Dekompensation	EKT (Elektrokonvulsionstherapie) als ärztliche Zwangsmaßnahme, anschließend freiwillige Fortführung	Rasche Remission, Wiederherstellung Einwilligungsfähigkeit

Psychopathologisch zeigte sich eine floride schizomanische Symptomatik. Affektiv imponierten eine maniforme Antriebssteigerung, psychomotorische Unruhe, Logorrhö und Affektinkontinenz. Psychotisch bestanden Bedrohungs- und Vergiftungswahn, formalgedankliche Zerfahrenheit bis hin zu Schizophasie. Wegen desorganisierten Verhaltens und Fehlhandlungen bestand erhebliche Eigen- und Fremdgefährdung.

Krankheitsbedingt war die behandlungsbezogene Einwilligungsfähigkeit aufgehoben, sodass keine selbstbestimmte, informierte Therapieentscheidung möglich war [1]. Die Medikation erfolgte nach wiederholter Ablehnung zeitweise als betreuungsgerichtlich genehmigte ärztliche Zwangsmaßnahme. Trotz mehrfacher Anpassungen (u. a. Risperidon bis 6 mg/Tag, Olanzapin bis 25 mg/Tag, Flupentixol Depot bis 50 mg/14 Tage, Pimozid bis 16 mg/Tag, Valproat bis 1200 mg/Tag, Diazepam bis 50 mg/Tag) zeigte sich keine klinische Stabilisierung. Aus der therapieresistenten Symptomatik resultierte ein erheblicher sozialmedizinischer Entscheidungsdruck bezüglich der weiteren Behandlungs- und Wohnperspektive, da eine Rückkehr nach Hause nicht möglich war und stattdessen eine geschützte Wohnform mit möglicher Aufgabe der bisherigen Wohnung erwogen wurde.

Leitliniengemäß und gemäß der Stellungnahme deutschsprachiger Fachgesellschaften wurde eine EKT als Behandlungsoption erwogen [2, 3], die die Patientin – wie in allen Voraufenthalten – psychotisch motiviert ablehnte. Nach betreuungsgerichtlicher Genehmigung wurde die EKT (unilateral rechts, Ladung 55 %) zunächst gegen den natürlichen Willen begonnen. Die Patientin protestierte initial lautstark und äußerte wiederholt Ablehnung. Die Maßnahmen konnten jedoch unter deeskalierender Ansprache mit hoher Personalpräsenz und permanenter Begleitung durchgeführt werden; unmittelbarer körperlicher Zwang oder Fixierung waren nicht erforderlich. Die EKT wurde insgesamt gut vertragen. Leichte Nebenwirkungen traten in Form vorübergehender Orientierungsstörung und mnestischer Defizite auf. Bereits nach 2 Sitzungen besserten sich die formalen Denkstörungen mit Wegfall der Schizophasie sowie die psychomotorische Erregung deutlich. Nach 3 Sitzungen entwickelte die Patientin Krankheitseinsicht und stimmte der Fortführung der Behandlung zu. Nach der EKT-Serie (12 Sitzungen) waren Wahn und formale Denkstörungen vollständig remittiert, woraufhin eine postpsychotische depressive Symptomatik mit Antriebsmangel, Insuffizienzgefühlen sowie gedrückter Stimmung und fehlender affektiver Auslenkbarkeit in den Vordergrund trat. Die Patientin führte dies auf die Medikation zurück, was psychoedukativ bearbeitet wurde.

Die Entlassung erfolgte unter Erhaltungs-EKT und Medikation (Amisulprid 400 mg/Tag, Olanzapin 20 mg/Tag) mit ambulanter Unterstützung in die eigene Wohnung. Infolge einer auf Wunsch der Patientin vorgenommenen Olanzapinreduktion kam es zur erneuten Dekompensation, die wiederum gut auf eine EKT-Serie (unilateral rechts, Ladung 80 %) ansprach. Auch diese wurde initial psychotisch motiviert abgelehnt und musste zunächst erneut gegen den natürlichen Willen der Patientin durchgeführt werden. Eine Behandlungsvereinbarung für zukünftige Episoden wurde nach Wiedererlangen der Krankheitseinsicht thematisiert, jedoch vor der erneuten Dekompensation nicht finalisiert.

## Diskussion

Der Fall zeigt, dass auch bei jahrzehntelang chronifizierter, pharmakotherapieresistenter schizoaffektiver Störung noch eine rasche, klinisch relevante Remission durch EKT möglich sein kann. Sein Beitrag liegt weniger im allgemeinen Nachweis der EKT-Wirksamkeit als in der Verlaufsbeobachtung eines langjährig ungünstigen schizoaffektiven Verlaufs.

Die Wirksamkeit der EKT ist gut belegt [4], auch bei Therapieresistenz [5, 6] und bei fehlender behandlungsbezogener Einwilligungsfähigkeit, wobei hohe Ansprechraten und teils eine Wiederherstellung der Entscheidungsfähigkeit beschrieben wurden [7, 8]. Auch aktuelle naturalistische Versorgungsdaten zeigen hohe klinische Verbesserungsraten bei Patienten mit Schizophrenie und schizoaffektiver Störung [9].

Gleichwohl bleibt die spezifische Evidenz für schizoaffektive Störungen begrenzt, da diese meist in gemischten psychotischen beziehungsweise affektiven Kohorten untersucht wurden [10, 11]. Der vorliegende Fall ergänzt die Literatur durch die Darstellung einer raschen EKT-Response nach jahrzehntelanger Erkrankungsdauer. Mindestens ein vergleichbarer Fall wurde bereits beschrieben [12]. Neben der Symptomremission war die Wiederherstellung der Einwilligungsfähigkeit entscheidend und unterstreicht das in der Literatur beschriebene Potenzial der EKT zur Wiedergewinnung von Autonomie [7, 8]. Im vorliegenden Fall veränderte sich dadurch zudem die Versorgungsperspektive: Eine langfristige geschützte Unterbringung konnte vermieden und eine Entlassung in die eigene Wohnung mit ambulanter Unterstützung ermöglicht werden. Juristisch bleibt zwischen der medizinischen Indikation einer EKT und den Voraussetzungen einer Behandlung gegen den natürlichen Willen zu unterscheiden. Die Deutsche Gesellschaft für Psychiatrie und Psychotherapie, Psychosomatik und Nervenheilkunde e. V. (DGPPN) betont, dass für die EKT aus medizinethischer Sicht keine strengeren Maßstäbe gelten sollten als für andere Verfahren und die mögliche Wiederherstellung der Einwilligungsfähigkeit besonders zu berücksichtigen ist; entsprechend stellt der Bundesgerichtshof an eine EKT gegen den natürlichen Willen besonders hohe Anforderungen und setzt stets eine sorgfältige Einzelfallprüfung voraus [13].

Der Fall unterstreicht zudem die Bedeutung einer frühzeitigen antizipierten Behandlungsplanung nach Wiedererlangung der Einwilligungsfähigkeit, beispielsweise im Rahmen von Behandlungsvereinbarungen oder Patientenverfügungen.

Insgesamt spricht der Verlauf dafür, die EKT bei schwerer, chronifizierter schizoaffektiver Störung auch nach jahrzehntelang ungünstigem Verlauf als therapeutische Option zu prüfen.

## Data Availability

Alle der vorliegenden Arbeit zugrunde liegenden Daten sind in diesem Artikel enthalten.
